# Quality and Oral Processing Characteristics of Traditional Serbian Ćevap Influenced by Game Meat

**DOI:** 10.3390/foods12102070

**Published:** 2023-05-21

**Authors:** Ilija Djekic, Slavisa Stajic, Bozidar Udovicki, Caba Siladji, Vesna Djordjevic, Nino Terjung, Volker Heinz, Igor Tomasevic

**Affiliations:** 1 Faculty of Agriculture, University of Belgrade, Nemanjina 6, 11080 Belgrade, Serbia; stajic@agrif.bg.ac.rs (S.S.); bozidar.udovicki@agrif.bg.ac.rs (B.U.); 2 Institute of Meat Hygiene and Technology, Kaćanskog 13, 11040 Belgrade, Serbia; caba.siladji@inmes.rs (C.S.); vesna.djordjevic@inmes.rs (V.D.); 3 German Institute of Food Technologies (DIL), 49610 Quackenbruck, Germany; n.terjung@dil-tec.de (N.T.); v.heinz@dil-ev.de (V.H.)

**Keywords:** wild boar meat, deer meat, sustainable game meat diet, sensory analysis, oral processing

## Abstract

This study analyzes the influence of two different types of game meat (deer and wild boar) in relation to quality characteristics and oral processing attributes of skinless sausage. The goal of this study was to compare grilled game-meat-based “ćevap” with conventional pork-meat-based samples. Research comprised of color analysis, evaluation of textural components, testing degree of difference, temporal dominance of sensations, calculation of main oral processing attributes, and examination of particle size distribution. The results show that oral processing attributes are similar in between samples and concur with results of the pork-based sample. This confirms the working hypothesis that it is possible to make game-meat-based “ćevap” fully comparable with conventional pork meat products. In parallel, color and flavor characteristics are influenced by the type of game meat in the sample. Most of the dominant sensory attributes that occurred during mastication were game meat flavor and juiciness.

## 1. Introduction

The consumption of meat worldwide shows an increase in overall meat production and increase in consumption per capita [1]. The rationale for meat consumption spreads from its nutritional values to sensory preferences [2,3]. However, meat is recognized as the type of food with the greatest environmental impact throughout the food supply chain [4]. Its production at the beginning and consumption at the end of the value chain affect the entire environment with excessive water consumption, pollution of soil and water, climate change, and loss of biodiversity [5,6]. Recognized routes to sustainable diets are through avoiding meat (and animal-origin food), development of alternatives such as plant-based meat analogues [7], or substituting traditional meat with insect-based proteins [8]. One of the main ideas behind meat alternatives is the potential of imitating textural, sensorial, and oral processing properties of conventional meat [9]. The shift in preferences towards these types of products needs to be sustainable and healthy [10].

Several authors identify wild game meat as a promising, popular sustainable alternative [11]. Nogueira and Nogueira-Filho [12] specify that native wild fauna provide both nutritional and sustainable perspectives. The game meat industry has the potential to support UN sustainable development goals in some African countries by combating hunger and food insecurity [13]. From a luxury food perspective, meals based on game meat are recognized as a combination of sustainable and healthy food [14]. In the retail sector, the promotion of game meat as a climate-friendly product is another advantage of this type of meat [15]. In spite of game meat advantages, Demartini et al. [16] emphasize that game meat consumption still receives less attention from the academia compared to traditional meat, despite its nutritional and sustainable benefits. In the U.S., game meat consumption is about 3% of the total consumption of all types of meat per annum. In Serbia, this type of meat is consumed less than once a month, where wild boar and deer meat prevail [17]. However, it may be assumed that the total share is slightly above that, mainly due to unreported consumption of small game meat. In line with these predictions, the consumption of wild game may avoid two billion kilograms of CO_2_ emissions, or even more [18].

Consumers worldwide express more and more interest in consuming game meat, and this trend is increasing every year, holding the reputation of exotic and luxurious meat [19,20]. Their appreciation for game meat is attributed to its nutritional value, as well as for its taste, aroma, and natural origin [21,22,23,24]. This type of meat is recognized as being healthier and highly nutritious, with its specific taste. In parallel, there is a belief that this type of meat comes from animals free of hormones and drugs. Finally, it is a good source of both micro- and macronutrients [25]. A cross-European survey on consumer perception towards game meat revealed that they favor its health benefits, nutritional characteristics, taste, overall quality, and potential to be organic [17].

As opposed to traditional meat products, consumers with higher health consciousness strive for meat with lower levels of fat and cholesterol, where game meat is recognized as a suitable option [26]. Game meat’s total fat content can be considered low, as it is generally around 5%, with some species achieving levels below 0.5% [25]. Additionally, structural lipids with a preferable fatty acid profile prevail in game meat [21,26,27]. On the contrary, protein content is generally higher than 20%, with some species reaching above 25% [25].

In Serbia, pork meat is the type of meat that is most consumed. Minced meat grilled products are one of the most popular types of food [28]. This type of meat product is also easy to prepare at home [29]. However, despite its nutritional benefits, consuming (grilled) meat has lately been associated with health issues such as cancer and diabetes [30]. In parallel, the discussion of its advantages and disadvantages covers the environmental dimension criticizing pork meat production and consumption [31]. One of the central extrinsic quality cues behind choosing minced meat is meat type, although animal welfare and environment concern are also important [29].

Ćevap (pronounced /t͡ɕěʋaːp/), is a minced meat product made of different types of meat (mostly pork and beef) and formed into a cylindrical shape (app. Ø 2 cm, 6–8 cm in length) that is often consumed as a local and national dish [28]. To endorse game meat, it is important to promote its highly ranked attributes associated with both intrinsic (such as sensory attributes and health benefits) and extrinsic quality features such as its sustainability potential [17,32]. In parallel, the latest studies show that in developing new products, as well as quality, oral processing plays an important role [33].

The working hypothesis of this research was that it is possible to develop a game-meat-based ćevap with characteristics like those of conventional pork-meat-based product. In that sense, the main objective of this research was to characterize quality and oral processing characteristics of grilled game-meat-based skinless sausage. In parallel, this study analyzed the intertwining of oral processing and quality perceptions of the products.

## 2. Materials and Methods

### 2.1. Ćevap Preparation

Deer and wild boar meats (round muscles) were purchased from a local meat store while pork meat and pork solid fat tissue (SFT) were purchased from the local green market (Belgrade, Serbia). The visible fat and connective tissue were trimmed off the pork meat. Average moisture, protein, and fat contents of the meat used for ćevap preparation are presented in Table 1. The meat was ground through an 8 mm sieve plate (82H, Laska, Traun, Austria), salted with NaCl (2% *w*/*w*), and left overnight at 4 °C. Samples were prepared according to a common industrial recipe as follows: 87.5% meat mixture, 11.0% ice water, 0.55% dextrose, 0.35% sodium bicarbonate, 0.4% sodium ascorbate, and 0.2% NaCl. Ćevap meat mixture varieties were prepared as follows: 40% deer meat, 40% wild boar meat, 20% SFT—sample “A”; 20% deer meat, 60% wild boar meat, 20% SFT—sample “B”; 60% deer meat, 20% wild boar meat, 20% SFT—sample “D”; and 80% pork meat, 20% SFT—sample “C”—control (Table 1). After mixing, the mixture was ground through a 5 mm sieve. Completed ćevap mixtures were put in the manual sausage feeder and pushed through a 25 mm funnel. The length of the finished products was set at approximately 6 cm. After preparation and before grilling, specimens were left to rest for eight hours at 4 °C.

The products were grilled using a Tefal grill (OptiGrill+) until the thermal center temperature of 72 °C was reached, as proposed by Ngapo et al. [34] and Djekic et al. [35]. A digital thermometer was used for temperature control during grilling (Trotec GmbH-Model BT20, Heinsberg, Germany).

### 2.2. Quality Characterization

Basic physicochemical characteristics. On the production day, the moisture [36], protein [37], and fat [38] contents of the samples were determined. The grilling loss was calculated by measuring the weight differences before and after treatment with the use of analytical balance (OHAUS Adventurer—Model AR2140, Parsippany, NJ 07054 USA), in line with Jeong et al. [39]. Measurement was performed using data from seven samples of each type of ćevap. Three individual specimens from each variant were used for pH measurement using a pH meter Testo 206 pH2 (Lenzkirich, Germany) equipped with a penetration probe. Before measurement, the pH meter was calibrated using standard buffer solutions at pH 4 and pH 7.

Color measurement. The color of the samples (both fresh and grilled) was measured with a Computer Vision System (CVS), as described in Tomasevic et al. [40]. A total of 10 measurements in 3 replicates from each sample were captured. Values were presented in CIELAB coordinates (L*, a*, and b*). The total color differences were measured, as presented in Equation (1) [41]:

(1) $$\Delta E=\sqrt{{\left(a_{C}{}^{*}-a_{G}^{*}\right)}^{2}+{\left(b_{C}-b_{G}^{*}\right)}^{2}+{\left(L_{C}{}^{*}-L_{G}^{*}\right)}^{2}}$$

Values for *a_C_, b_C_*, and *L_C_* were obtained from sample “C”, which served as a control. Values for *a_G_, b_G_*, and *L_G_* were obtained from the samples with game meat (samples “A”, “B”, and “D”).

Instrumental texture analysis. Texture profile analysis (TPA) was performed using TA.XT plus Texture Analyzer (Stable Micro System, Godalming Surrey GU7 1YL, United Kingdom ). Six grilled specimens from each sample were tested in two replicates using a sample size cylinder: height 25 mm, diameter 35 mm. For the tests, parameters were as follows: pre-test speed (180 mm/min), test speed (60 mm/min), post-test speed (80 mm/min), target mode distance (50%), probe selection (P/100), sample shape (cylindrical), and time between cycles (10 s). A digital caliper was used for measuring the dimension of the samples. A thin-bladed sharp knife was used for sample preparation [42].

### 2.3. Sensory Analysis

Sensory panel setup. The panel consisted of eight panelists (four male and four female) with experience in previous sensory studies. They were of good general health condition with a body mass index between 18 and 25 kg/m^2^, as recommended by Forde et al. [43]. The same panel was used for oral processing characterization as they did not report any dental problems. Four 2 h training sessions in the period of two weeks were conducted to train the panelists for the sensory and oral processing methods used, as suggested by Djekic et al. [44].

Degree of difference testing was employed to distinguish the size of the difference from the control sample focused on measuring the differences in key sensory attributes of game meat samples. Similar to the research by Stajić et al. [45], an intensity scale with nine points was used with the following attributes: juiciness (1 = dry, 5 = control “C“, and 9 = very juicy); hardness (1 = soft, 5 = “C”, and 9 = firm); meat taste (1 = not enough, 5 = “C”, and 9 = too much); and meat odor (1 = not enough, 5 = “C”, and 9 = too much). In parallel, the intensity of the game meat (compared to the control) was also evaluated using the scale from 5 to 9, as follows: game meat taste (5 = “C”—no game meat, and 9 = too intense); game meat odor (5 = “C”—no game meat, and 9 = too intense). Testing was performed in two replicates.

Temporal Dominance of Sensations (TDS). Prior to initiating TDS, all panelists became familiar with the following attributes: meat flavor, game meat flavor, firm, juicy, dry, and soft, extracted from previous research [35,46,47]. Upon becoming accustomed to the selected sensations, TDS was performed as outlined in Djekic et al. [35]. The panel was asked to select a dominant attribute, pointing out that a sensation could be selected several times. Serving was performed randomly. Panelists had the opportunity to consume white bread and drink tap water to remove potential aftertastes. TDS was performed in two replicates.

The dominance rate, plotting, and standardization of the time scale of mastication were performed as outlined in [35,48]. In parallel, a chance level line was added, calculated according to Pineau et al. [49].

### 2.4. Oral Processing Characterization

Mastication parameters. To collect data on oral processing, grilled samples were served to panelists. The mass of each sample was measured using a technical balance with a 0.01 g accuracy. A digital camera, placed 30 cm from the panelists, captured their upper body during mastication [43]. Panelists were instructed to look straight into the camera during mastication. When swallowing, they were asked to raise their hand, emphasizing they could swallow several times.

All video clips were examined (using a stopwatch), and two main features were captured: the number of chews and the mastication duration [50,51]. Further calculation revealed chewing rate, eating rate, and number of swallows [43,52]. All samples (control and three combinations of game meat) were provided to the panelists in two replications.

Particle size distribution. To analyze food breakage during mastication, boluses from each panelist were gathered after 12 strokes and just before swallowing. The first step was to rinse them with distilled water on filter paper. The next step was to spread them with special care to avoid damage. The third step was to take photos of the spread particles. Finally, all images were processed using ImageJ software. This enabled counting the number of particles, calculating particle surfaces, and analysis of particle size distribution [53]. This testing was completed in two replicates.

Statistical processing. One-way ANOVA and Tukey’s HSD post hoc test were employed with the level of statistical significance set at 0.05.

## 3. Results and Discussion

### 3.1. Quality-Related Results

Moisture, protein, and fat contents showed no statistical differences (*p* > 0.05), which was expected, considering the similar contents of these parameters to meat used for ćevap preparation (Table 1). The pH values of raw ćevap were similar except in sample “D”, which mostly consisted of deer meat. The pH values were somewhat higher compared to similar products—beef and pork burgers [54,55]. Though sample “D” had the lowest pH value, the values of grilling loss were significantly lower compared to other treatments. However, this did not affect the basic chemical composition.

The color of the samples shows that there are statistical differences between them (Table 2). Lightness (L*) values were the highest for the control sample for all three types of samples (fresh meat, fresh fat, grilled), while the samples with game meat showed lower values (*p* < 0.05). Redness (positive a* values) were the highest for fresh meat control sample, but lowest for fresh fat parts and grilled intersections. The opposite trend in redness was observed on the samples with game meat. Yellowness (positive b* values) were similar for all four fresh meat samples, but lowest for the control samples (fresh fat and grilled intersection). Besides the control sample, a statistically significant difference was observed for most of the color parameters for sample “B” compared to the other samples. It was expected that game meat affects color in terms of its darkening [22]. The total color difference values of fresh samples show that the color of meat parts has a higher degree of difference compared to fat parts (values above 10 compared to values below 10). Regarding grilled parts, values of ΔΕ were around 10. When the values of ΔE were within a range from 2 to 10, there was a perceptible difference, while values above 10 were considered significant visible differences [40].

The color of meat is very important for consumers’ perception of meat quality [22,56]. Although color is not a reliable indicator regarding quality, it is one of the main parameters behind purchasing decisions [57]. Game meat and meat products are often perceived as being very dark in color [58,59]. A dark meat color is attributed to higher ante-mortem muscle activities and myoglobin content and ante-mortem stress (increased red muscle fibers) [21,60].

Textural values (Table 3) showed that for four of the parameters, there was no statistically significant difference between the samples (*p* > 0.05). Springiness, cohesiveness, and resilience were the parameters with the highest differences (*p* < 0.05) between sample “B” and control “C”. Chewiness was reported as being the highest (*p* > 0.05) in the control sample, related to crust formation during the grilling of pork meat, resulting in harder and chewier meat [35,39]. The highest level of grilling loss was obtained for sample “B”, and the lowest for sample “D” (*p* < 0.05), with all results below 25%, as reported by Djekic et al. [35] with grilled pork ham.

**Figure 1:**
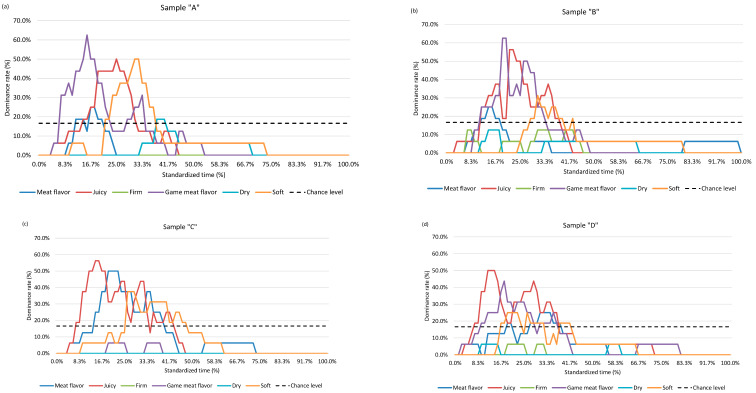
Temporal dominance sensations (TDS) curve for four samples. Defined time axis from *t* = 0 (first bite—0%) to *t* = 1 (swallowing—100%). Attributes are shown in different colors. Legend: Sample “A”—40% deer meat, 40% wild boar meat, 20% pork solid fat tissue (SFT)—subfigure (**a**); Sample “B”—20% deer meat, 60% wild boar meat, 20% SFT—subfigure (**b**); Sample “C”—80% pork meat, 20% SFT—control—subfigure (**c**); Sample “D”—60% deer meat, 20% wild boar meat, 20% SFT—subfigure (**d**).

Figure 1 shows the proportion of each analyzed sensory attribute recorded during the consumption of the four samples. For sample “A”, game meat flavor, juiciness (at the beginning of mastication), and softness (during the first half of mastication) were the predominant attributes. A similar pattern was observed for sample “C” with the mixture of meat flavor, juiciness, and softness. Samples “B” and “D” showed an intertwining mix of game meat flavor and juiciness in the first half of mastication. For all four samples, during the second half of the mastication period, sensory attributes significantly faded.

When compared with pork meat, wild boar has microstructural differences that affect its juiciness and tenderness [47]. The flavor of game meat is considered as a complex but important sensory attribute influenced by many factors such as texture, presence of different compounds, and juiciness, where the intensity of game meat odor and aroma prevail [22]. Game meat flavor or “wild” flavors in meat are most likely contributed by natural grazing, which may partly be reflected in the effects of fatty acid composition [61]. This was confirmed in the study on deer meat, where flavor was dominated by the diets of animals before slaughtering [62]. Its flavor is considered “stronger” when compared to meat from beef, pork, and/or lamb. However, when it comes to wild boar flavor perception, volatile compounds in pork and wild boar meat are similar [63], and the quantity of the volatiles that are released during mastication make a difference [47]. Swanson and Penfield [64] consider higher polyunsaturated fatty acid percentages in game meat as the main cause for such taste. The quality of wild boar meat is in correlation with animal age [65], while for deer meat, sex and hunting period make the difference [66].

**Figure 2:**
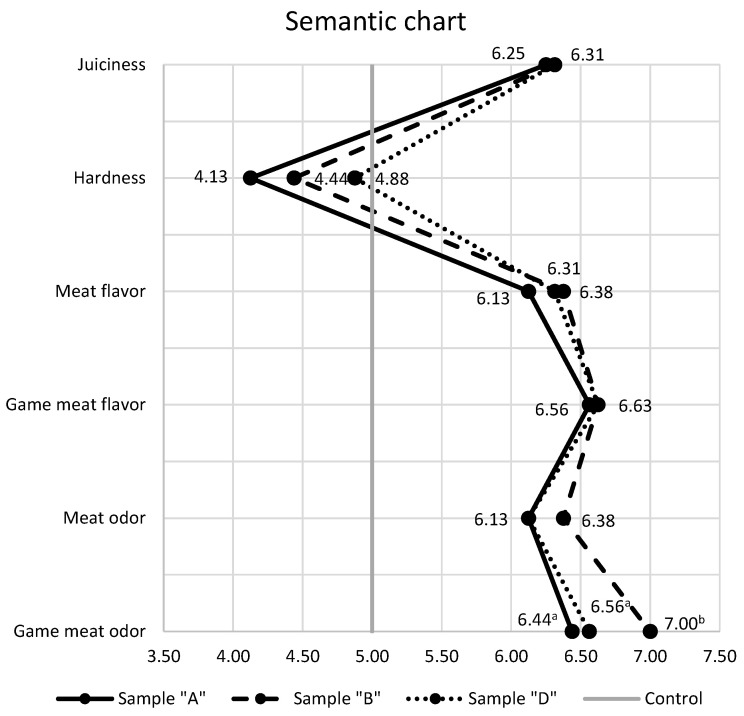
Semantic differential chart. Mean values within the same row with the different superscripts differ significantly (*p* < 0.05). For all sensory attributes, a value equal to 5 corresponds to the control sample (Sample “C”). Legend: Sample “A”—40% deer meat, 40% wild boar meat, 20% pork solid fat tissue (SFT); Sample “B”—20% deer meat, 60% wild boar meat, 20% SFT; Sample “D”—60% deer meat, 20% wild boar meat, 20% SFT; Sample “C”—80% pork meat, 20% SFT—control.

Semantic chart (Figure 2) shows that compared to the control sample, all sensory attributes follow a similar pattern, with no statistically significant difference (*p* > 0.05).

### 3.2. Oral Processing Results

Table 4 presents all the calculated oral processing parameters. All four samples had similar results with no statistically significant difference (*p* > 0.05), meaning that all samples with game meat showed no difference compared to the control and that average consumers would not observe any difference. This result aligns with the working hypothesis that samples with game meat have similar characteristics to often-consumed pork-based ćevap. The latest study on the perception of consumers to oral processing characteristics performed by Djekic et al. [33] revealed the importance of the number of chews and eating rate associated with different types of food.

The average number of chews was between 64 and 67, with a total exposure time of around 45 s. The chewing rate in the range of 1.38 chew/s–1.48 chew/s concurs with the chewing rate of pork meat (1.52 chew/s) as presented in Djekic et al. [35] or Farooq and Sazonov [67], with average chewing rate of 1.53 chew/s.

**Figure 3:**
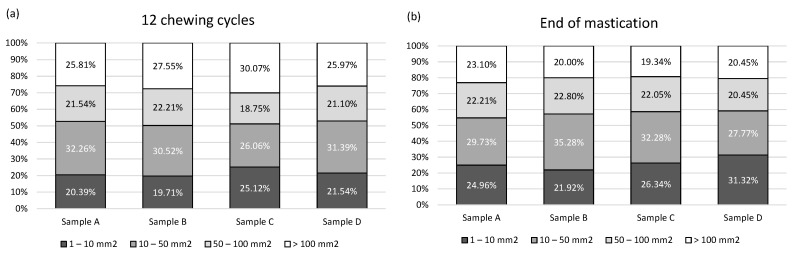
Percentage of area occupied by particles of size: 1–10 mm^2^ (light grey), 10–50 mm^2^ (dark grey), 50–100 mm^2^ (black color), and >100 mm^2^ (white color), depending on the sample after 12 chewing cycles—subfigure (**a**) and at the end of mastication—subfigure (**b**). Legend: Sample “A”—40% deer meat, 40% wild boar meat, 20% pork solid fat tissue (SFT); Sample “B”—20% deer meat, 60% wild boar meat, 20% SFT; Sample “D”—60% deer meat, 20% wild boar meat, 20% SFT; Sample “C”—80% pork meat, 20% SFT —control.

Particle size distribution during mastication and before swallowing are related to mechanical food properties [53]. Figure 3 depicts the percentage of the area occupied by particles of different sizes (after 12 chews and before swallowing). After 12 chewing cycles, boluses for all four samples had below 30% of large particles (>100 mm^2^) and around 50% of the area with particles smaller than 50 mm^2^. At the end of mastication, boluses consisted of below 20% of large particles (>100 mm^2^) and below 60% of the area was occupied by particles smaller than 50 mm^2^. This confirms that the proportion of bigger particles decreases over time [35,53]. Food breakdown is defined as an “attractive” oral processing characteristic resulting in customer satisfaction [33]. Our results confirm that samples with game meat align with the food breakdown pattern of often-consumed pork-meat-based ćevap.

**Figure 4:**
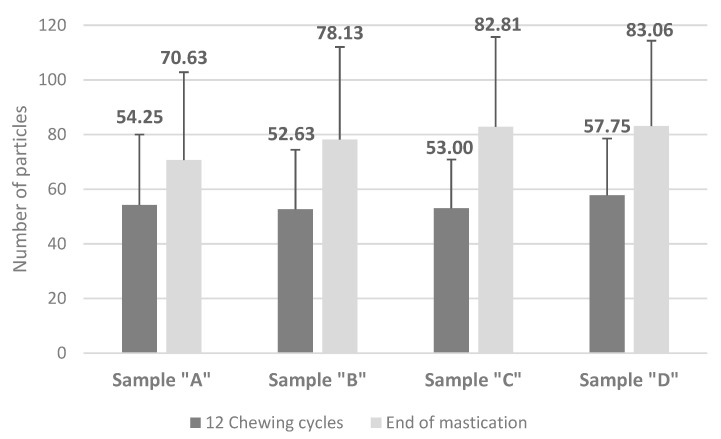
Number of particles of samples obtained in vivo after 12 chewing cycles (dark grey bars) and at the end of mastication (light grey bars). Legend: Sample “A”—40% deer meat, 40% wild boar meat, 20% pork solid fat tissue (SFT); Sample “B”—20% deer meat, 60% wild boar meat, 20% SFT; Sample “D”—60% deer meat, 20% wild boar meat, 20% SFT; Sample “C”—80% pork meat, 20% SFT —control.

The number of particles (Figure 4) in the bolus increased with the number of chewing strokes, from below 60 (after 12 chewing strokes) to above 70 at the end of mastication. The lowest number of particles at the end of mastication was counted from sample “A”, and the highest from sample “D”.

## 4. Conclusions

This study shows the potential of using game meat as a sustainable substitute to pork meat, obtaining similar characteristics. All oral processing characteristics of game meat samples align with the control (pork) samples, with no adverse effects. Opposed to this, quality characteristics are fully dependent on the presence of game meat, mainly in terms of color and flavor. This comprehensive study may be helpful for both academia and the meat sector in developing more sustainable meat-based products.

This research is one of the first to investigate the oral processing attributes of game-meat-based products and their inter-link with sensory and quality characteristics. A certain limitation of the study is that consumers were not included in the study.

## Figures and Tables

**Table 1:** Basic physicochemical values of the samples.

Parameter	Sample “A”	Sample “B”	Sample “C”	Sample “D”
Meat mixtures	40% deer meat; 40% wild boar meat; 20% SFT	20% deer meat; 60% wild boar meat; 20% SFT	80% pork meat; 20% SFT	60% deer meat; 20% wild boar meat; 20% SFT
Moisture content (%)	65.44 ± 1.04 ^a^	65.11 ± 0.92 ^a^	65.35 ± 1.38 ^a^	65.41 ± 1.95 ^a^
Protein content (%)	16.70 ± 0.23 ^a^	16.45 ± 1.40 ^a^	15.53 ± 1.00 ^a^	16.21 ± 0.46 ^a^
Fat content (%)	16.14 ± 0.80 ^a^	16.01 ± 1.54 ^a^	16.67 ± 0.26 ^a^	16.26 ± 0.57 ^a^
pH value	6.74 ± 0.07 ^a^	6.69 ± 0.09 ^a^	6.83 ± 0.04 ^a^	6.34 ± 0.06 ^b^

Values (means ± standard deviation) in the same row with different superscripts are significantly different (*p* < 0.05). SFT—pork solid fat tissue.

**Table 2:** The effects of different combinations of game meat on the color properties of the samples.

Fresh Samples	Sample “A”	Sample “B”	Sample “C”	Sample “D”
	Meat Part			
L*	37.7 ± 7.5 ^a^	41.1 ± 5.5 ^a^	50.7 ± 4.7 ^b^	41.0 ± 4.4 ^a^
a*	31.8 ± 4.3 ^a^	32.4 ± 2.9 ^a,b^	36.0 ± 2.2 ^b^	31.3 ± 3.7 ^a^
b*	10.9 ± 2.0	10.4 ± 2.1	10.4 ± 1.4	11.2 ± 4.5
**ΔΕ**	**14.8 ± 6.7**	**11.2 ± 4.5**		**12.1 ± 4.5**
	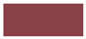	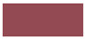	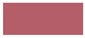	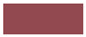
**Fresh samples**	**Fat part**			
L*	78.4 ± 3.5 ^a,b^	76.4 ± 5.8 ^a^	81.7 ± 2.9 ^b^	79.7 ± 4.3 ^a,b^
a*	7.7 ± 1.9 ^a^	11.4 ± 3.9 ^b^	6.8 ± 1.7 ^a^	7.4 ± 2.0 ^a^
b*	2.1 ± 1.3 ^a^	4.1 ± 2.1 ^b^	0.4 ± 1.1 ^a^	1.9 ± 1.2 ^a^
**ΔΕ**	**4.7 ± 3.0**	**9.1 ± 5.5**		**4.3 ± 3.3**
	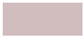	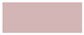	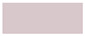	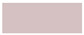
**Grilled samples**	**Intersection**			
L*	60.9 ± 2.7 ^a^	60.3 ± 2.1 ^a^	70.2 ± 2.4 ^b^	61.7 ± 4.5 ^a^
a*	15.4 ± 1.4 ^a^	15.1 ± 1.2 ^a^	12.6 ± 1.4 ^b^	14.5 ± 1.5 ^a^
b*	7.4 ± 1.2 ^a^	8.8 ± 1.0 ^b^	6.8 ± 1.2 ^a^	7.2 ± 0.4 ^a^
**ΔΕ**	**9.8 ± 2.9**	**10.5 ± 2.2**		**8.8 ± 4.6**
	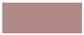	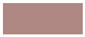	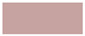	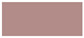

Means of 10 × 3 replications ± standard deviation. Items denoted with different letters are significantly different at the level of 5%. Legend: Sample “A”—40% deer meat, 40% wild boar meat, 20% pork solid fat tissue (SFT); Sample “B”—20% deer meat, 60% wild boar meat, 20% SFT; Sample “D”—60% deer meat, 20% wild boar meat, 20% SFT; Sample “C”—80% pork meat, 20% SFT—control.

**Table 3:** The effects of different combinations of game meat on instrumental textural properties of the samples.

Parameter	Sample “A”	Sample “B”	Sample “C”	Sample “D”
Hardness [n]	36.53 ± 2.21	35.76 ± 3.43	35.09 ± 1.97	35.28 ± 4.84
Adhesiveness [g·s]	−0.87 ± 1.30	−6.49 ± 16.80	−4.30 ± 4.51	−0.78 ± 0.81
Springiness	0.90 ± 0.01 ^a,b^	0.92 ± 0.02 ^a^	0.89 ± 0.02 ^b^	0.91 ± 0.03 ^a,b^
Cohesiveness	0.40 ± 0.05 ^a^	0.38 ± 0.02 ^a^	0.45 ± 0.07 ^b^	0.39 ± 0.04 ^a^
Gumminess	14.53 ± 2.29	13.52 ± 1.43	15.88 ± 2.51	13.81 ± 2.37
Chewiness [g]	13.10 ± 2.06	12.43 ± 1.40	14.19 ± 2.07	12.51 ± 2.03
Resilience	0.18 ± 0.03 ^a,b^	0.16 ± 0.02 ^a^	0.20 ± 0.04 ^b^	0.17 ± 0.02 ^a,b^
Cooking loss (%)	17.95 ± 1.54 ^a^	21.06 ± 2.15 ^b^	17.99 ± 1.16 ^a^	14.23 ± 1.98 ^c^

Note: Items denoted with different letters are significantly different at the level of 5%. Legend: Sample “A”—40% deer meat, 40% wild boar meat, 20% pork solid fat tissue (SFT); Sample “B”—20% deer meat, 60% wild boar meat, 20% SFT; Sample “D”—60% deer meat, 20% wild boar meat, 20% SFT; Sample “C”—80% pork meat, 20% SFT—control.

**Table 4:** Summary of the oral processing behavior parameters for the samples.

Parameter	Sample “A”	Sample “B”	Sample “C”	Sample “D”
Number of chews	64.63 ± 28.77	65.06 ± 22.30	67.13 ± 20.40	65.63 ± 21.97
Total exposure time [s]	45.19 ± 25.11	46.81 ± 28.01	45.94 ± 26.03	43.88 ± 26.19
Number of swallows	2.63 ± 0.81	2.69 ± 0.70	2.44 ± 0.51	2.31 ± 0.60
Mass of chewing sample [g]	19.38 ± 1.68	20.08 ± 1.69	20.62 ± 2.22	20.15 ± 1.99
Chewing rate [chew/s]	1.41 ± 0.28	1.38 ± 0.26	1.45 ± 0.22	1.48 ± 0.23
Eating rate [g/s]	0.52 ± 0.19	0.52 ± 0.17	0.54 ± 0.20	0.58 ± 0.27
Number of chews per gram [chew/g]	3.37 ± 2.08	3.29 ± 2.30	3.24 ± 1.84	3.21 ± 1.96
Chewing cycle duration [s/chew]	0.74 ± 0.15	0.75 ± 0.14	0.71 ± 0.10	0.69 ± 0.10

Legend: Sample “A”—40% deer meat, 40% wild boar meat, 20% pork solid fat tissue (SFT); Sample “B”—20% deer meat, 60% wild boar meat, 20% SFT; Sample “D”—60% deer meat, 20% wild boar meat, 20% SFT; Sample “C”—80% pork meat, 20% SFT—control.

## Data Availability

The data presented in this study are available on request from the corresponding author.
